# Alpha-enolase (ENO1), identified as an antigen to monoclonal antibody 12C7, promotes the self-renewal and malignant phenotype of lung cancer stem cells by AMPK/mTOR pathway

**DOI:** 10.1186/s13287-021-02160-9

**Published:** 2021-02-12

**Authors:** Xiong Shu, Kai-Yue Cao, Hui-Qi Liu, Long Yu, Li-Xin Sun, Zhi-Hua Yang, Cheng-Ai Wu, Yu-Liang Ran

**Affiliations:** 1 Laboratory of Molecular Orthopaedics, Beijing Research Institute of Orthopaedics and Traumatology, Beijing JiShuiTan Hospital, No. 31 Xinjiekou E Road, Xicheng, Beijing, 100035 People’s Republic of China; 2 Department of Pathology, Tianjin First Central Hospital, Tianjin, People’s Republic of China; 3 Department of Basic Medical Science, Medical School of Qinghai University, Xining, People’s Republic of China; 4 State Key Laboratory of Molecular Oncology, National Cancer Center/National Clinical Research Center for Cancer/Cancer Hospital, Chinese Academy of Medical Sciences and Peking Union Medical College, No. 17 Panjiayuan Subdistrict, Chaoyang, Beijing, 100021 People’s Republic of China

**Keywords:** α-Enolase, Monoclonal antibody, Tumor-associated antigens, Cancer stem cells, Lung cancer

## Abstract

**Background:**

Tumor-associated antigens (TAAs) can be targeted in cancer therapy. We previously identified a monoclonal antibody (mAb) 12C7, which presented anti-tumor activity in lung cancer stem cells (LCSCs). Here, we aimed to identify the target antigen for 12C7 and confirm its role in LCSCs.

**Methods:**

Immunofluorescence was used for antigen localization. After targeted antigen purification by electrophoresis and immunoblot, the antigen was identified by LC-MALDI-TOF/TOF mass spectrometry, immunofluorescence, and immunoprecipitation. The overexpression or silence of ENO1 was induced by lentiviral transduction. Self-renewal, growth, and invasion of LCSCs were evaluated by sphere formation, colony formation, and invasion assay, respectively. High-throughput transcriptome sequencing (RNA-seq) and bioinformatics analysis were performed to analyze downstream targets and pathways of targeted antigen.

**Results:**

Targeted antigen showed a surface antigen expression pattern, and the 43–55 kDa protein band was identified as α-enolase (ENO1). Self-renewal, growth, and invasion abilities of LCSCs were remarkably inhibited by ENO1 downregulation, while enhanced by ENO1 upregulation. RNA-seq and bioinformatics analysis eventually screened 4 self-renewal-related and 6 invasion-related differentially expressed genes. GSEA analysis and qRT-PCR verified that ENO1 regulated self-renewal, invasion-related genes, and pathways. KEGG pathway analysis and immunoblot demonstrated that ENO1 inactivated AMPK pathway and activated mTOR pathway in LCSCs.

**Conclusions:**

ENO1 is identified as a targeted antigen of mAb 12C7 and plays a pivotal role in facilitating self-renewal, growth, and invasion of LCSCs. These findings provide a potent therapeutic target for the stem cell therapy for lung cancer and have potential to improve the anti-tumor activity of 12C7.

## Background

Lung cancer is a leading cause of cancer-related death worldwide [1, 2]. A recent investigation reported that approximately 1.8 million people are diagnosed with lung cancer, and 1.6 million people died of these diseases every year [3]. Despite remarkable advancements in the lung cancer therapy, including surgery, chemotherapy, and radiotherapy, especially targeted therapy, have been achieved in recent decades [4, 5], treatment outcomes remain unsatisfactory with the low 5-year survival rates, ranging from 4 to 17% [6, 7]. Therefore, the innovative diagnosis and treatment strategies are urgently needed to improve outcome for patient with lung cancer.

Growing evidences showed that cancer stem cells (CSCs) are defined as sub-population of tumor cells that possess the capacity to self-renew and high tumorigenicity, which are responsible for the tumor progression, spread, drug resistance, and recurrence [8]. Thus, targeting lung CSCs (LCSCs) may produce crucial advances in the innovative and more effective therapies. Fortunately, recent studies have proposed that LCSCs as therapeutic targets may provide powerful tools to improve the clinical outcome of patients with lung cancer [9]. Thus, here, we aimed to focus on the biological properties of LCSCs, including self-renewal, growth and invasion.

Currently, monoclonal antibody (mAb)-based therapeutics are standard methods in the cancer therapies [10, 11]. However, the antigen specificity could affect the characteristics of mAbs [10], thereby, the identification of target antigen and understand of downstream signaling pathway would be crucial for the therapeutic applications of mAb. In our previous study, we identified a functional antibody 12C7 that specifically target LCSCs and, more importantly, confirmed their inhibitory effects on biological properties of LCSCs both in vitro and in vivo [12]. However, their target antigen and the mechanism that drives the self-renewal of LCSCs are not fully understood [13]. Herein, the study was designed to identify the target antigen recognized by mAb 12C7 and investigate its function, expecting to find novel therapeutic targets for stem cell therapy and improving the anti-tumor activity of 12C7.

In present study, we identified α-enolase (ENO1) as an antigen of mAb 12C7 by using the western blot and immunoprecipitation, followed by the liquid chromatography-MALDI-tandem time-of-flight (LC-MALDI-TOF/TOF) analysis. Furthermore, we confirmed the tumorigenic role of ENO1 and proposed its potential mechanism by high-throughput transcriptome sequencing (RNA-seq) and bioinformatics analysis.

## Materials and methods

### Cell lines and cell culture

Human lung cancer A549 cell line was obtained from Laboratory of Antibody Engineering, Cancer Institute, Chinese Academy of Medical Sciences. Human lung adenocarcinoma SPCA-1 cell line was purchased from Type Culture Collection of the Chinese Academy of Sciences (Shanghai, China). Cells were maintained in RIPM 1640 medium supplementing with 10% fetal bovine serum (FBS), L-glutamine (2 mmol/L) penicillin (100 U/mL), and streptomycin (100 U/mL) in a humidified incubator containing 5% CO_2_ at 37 °C. Then, cells were subcultured using 0.2% trypsin and 0.1% EDTA when confluent (> 80% confluence).

Lung cancer stem cells (LCSCs) were enriched from A549 cells and SPCA-1 cells, respectively, using serum-free medium, as previously described [14]. Briefly, lung cells were collected after 48 h serial subcultivation and re-suspended in serum-free DMEM/F12 medium. Subsequently, the treated cells were seeded in culture bottle at a density of 2 × 10^4^ cells/mL and incubated at 37 °C with a 5% CO_2_ atmosphere. The medium containing growth factors was supplemented every 2 days. Finally, after the formation of the sphere (day 7), cells were harvested by centrifugation and used for the subsequent experiments.

### Immunofluorescence localization

The antigen localization was determined using immunofluorescence localization (live-cell and fixed-cell immunofluorescence staining). In brief, cells were grown in a 24-well plate for 48 h and washed with serum-free medium twice. For live-cell immunofluorescence, cells were incubated with corresponding primary antibodies to ENO1 (purified mAb 12C7 or commercial antibody to ENO1) for 1 h at room temperature and washed with phosphate buffer solution (PBS) with 1% bovine serum albumin (BSA). Cells were then fixed in 4% paraformaldehyde for 15 min.

For fixed-cell immunofluorescence, cells were firstly fixed in 4% paraformaldehyde for 15 min and then washed with PBS containing 1% BSA and 0.05% Tween-20 (Sigma-Aldrich, St. Louis, USA). Subsequently, cells were permeabilized with 0.02% TritonX-100 (Merck & Co., Inc., New Jersey, USA) and blocked with horse serum for 10 min. Next, cells were incubated with primary antibodies to ENO1 for 1 h at room temperature.

Subsequently, the following same treatments were performed for the live-cell and fixed-cell immunofluorescence. Cells were washed with PBS containing 1% BSA and 0.05% Tween-20, and incubated with secondary antibodies (Jackson ImmunoResearch, Pennsylvania, USA) for 30 min at room temperature. DAPI was used for nuclear staining. The cellular localization was visualized under a fluorescent microscope (Nikon, Japan) or laser scanning confocal microscope (Leica Microsystems, Wetzlar, Germany).

### Purification and identification of antigen recognized by mAb 12C7

The antigen recognized by mAb 12C7 was purified from SPCA-1 sphere cells using agarose G (GE, Boston, USA) and long-range SDS-PAGE. Briefly, SPCA-1 sphere cells were lysed with 200 μL RIPA buffer (Beyotime Biotechnology, Beijing, China) containing protease inhibitor cocktail (Roche, Basel, Switzerland). After centrifugation of cell lysates at 12,000 rpm, the protein supernatant was mixed with 20 μL agarose G, and centrifuged at 3000 rpm for 5 min to remove the non-specific adsorbed proteins. Then, the protein supernatant was added into 20 μL agarose G pre-conjugated with 127C antibody (10 μL) and incubated overnight at 4 °C. Subsequently, the mixture was centrifuged to remove supernatant. The proteins were eluted with PBS and separated on 10% SDS-PAGE. Next, the result of SDS-PAGE was visualized by Coomassie brilliant blue staining and Western blotting, respectively. Finally, the putative ENO1 band on SDS-PAGE was excised and then analyzed using LC-MALDI-TOF/TOF as described elsewhere [15]. The fragment sequences searched and analyzed using the Mascot database (http://www.matrixscience.com).

### Immunoprecipitation

Immunoprecipitation was performed to further identify the antigen. In brief, the agarose G was pre-incubated with 10 μg of commercial antibody to ENO1 (Abcam, Cambridge, UK), the purified mAb 12C7 or their corresponding negative control (NC), including normal mouse IgG and rabbit IgG. Then, cells were lysed with 200 μL RIPA buffer containing protease inhibitor cocktail. Total cell extracts were incubated with agarose G. Then, the protein were collected. Thereafter, the target protein was immunoprecipitated by incubating the supernatant with agarose-conjugated antibodies overnight at 4 °C. Samples were washed with PBS three times. Finally, efficiency of immunoprecipitation was confirmed by western blot using mAb 12C7 or commercial antibody to ENO1.

### Lentiviral production and transduction

Human ENO1 was inserted into the GV248 lentiviral vectors to silence ENO1, and into the GV358 lentiviral vectors to overexpress ENO1, respectively. The lentiviral vectors GV248, GV358, and corresponding NC (Lentiviruses CON238 and CON077) were obtained from Genechem (Shanghai, China). A549 and SPCA-1 cells were respectively seeded in the 6-well plate and further incubated for 24 h in complete medium. Then, lentiviral vectors were transfected into A549 and SPCA-1 cells at a multiplicity of infection (MOI) ranging from 1 to 100. To produce stably transfected cell lines, cells were cultured in the presence of puromycin. Cells were incubated for another 2 weeks and were harvested when the lentiviral transduction efficiency was greater than 90% as measured by the density of green fluorescent protein. Western blot was performed to confirm the expression of the target gene according to standard protocol. Then, cells were used for subsequent experiments.

### Sphere formation assay

Self-renewal ability was evaluated by sphere formation assay. LCSCs were enriched, respectively, from transfected A549 cells and SPCA-1 cells. Sphere cells were digested with tyrisin (Thermo Fisher scientific, USA) and plated onto a 24-well plate at a density of 500 cells/well in serum-free DMEM/F12 medium supplemented with methylcellulose (0.8%, Sigma-Aldrich, St. Louis, USA), epidermal growth factor (EGF, 20 ng/mL, Invitrogen) and basic fibroblast growth factor (bFGF, 20 ng/mL, Invitrogen), leukemia inhibitory factor (LIF, 10 ng/mL, Invitrogen), and B27 (1:50; Invitrogen). After incubation for 11 days, the number of spheres was counted under an inverted microscope (Nikon, Japan).

### Colony formation assay

Colony formation assay was performed to determine cell growth ability. Briefly, the transfected cells were seeded into a 24-well plate with and cultured for 14 days using the standard two-layer soft agar culture in a humidified incubator at 37 °C with a 5% CO_2_ atmosphere. Then, colonies were fixed with methanol for 30 min and stained with Giemsa stain for another 30 min. Stained colonies were visualized and manually counted under a microscope (Nikon, Japan).

### Cell invasion assay

Cell invasion ability was detected by matrigel-coated Transwell chamber. Briefly, 2 × 10^4^ cells/well transfected cells were plated in the upper chambers of 8 μm Transwells (Corning, USA) coated with matrigel (BD Biosciences, USA). Cells were maintained in the medium without serum or growth factors at 37 °C with 5% CO_2_. The medium containing 10% FBS was used as a chemoattractant in the lower chamber. After 24 h incubation, non-invading cells were removed from upper surface of matrigel. Cells that invaded the lower chamber were fixed with methanol and stained with 4,6-diamidino-2-phenylindole (DAPI). Finally, the stained cells were counted in three random fields per well using a light microscope (Nikon, Japan).

### RNA-seq and bioinformatics analysis

To identify the potential target of ENO1, the differentially expressed genes (DEGs) between the GV248-ShENO1B SPCA-1 cells and GV248-CON SPCA-1 cells (NC) were detected by RNA-seq. RNA-seq was performed by Hebei Jianhai Medical Laboratory (Shijiazhuang, Hebei, China). Briefly, total RNA was extracted and purified using Aurum™ total RNA Mini kit (Bio-Red, USA) following the manufacturer’s protocol. mRNA was isolated with Oligo (dT) beads from total RNA (10 μg) and fragmented into small pieces. Using these short fragments as templates, random hexamers were used to synthesize double-stranded cDNA using SuperScript Double-Stranded cDNA Synthesis Kit (Invitrogen, USA). Synthesized cDNA was subjected to end-repair, phosphorylation, 3′adenylation and ligation to sequencing adaptors using Illumina Truseq RNA Sample Preparation kit (Illumina, USA). Finally, PCR products were sequenced on Illumina HiSeq platform (Illumina, Inc., USA). Statistical software R and packages of limma were utilized to analyze DEGs. Gene ontology (GO) analysis, Kyoto Encyclopedia of Genes and Genomes (KEGG) pathway enrichment analysis, and Gene set enrichment analysis (GSEA) was performed for ENO1 and their putative targets to identify the potential pathways and networks involved in the ENO1-mediated lung cancer progression.

### Quantitative reverse transcription polymerase chain reaction (qRT-PCR)

The qRT-PCR analysis was performed to confirm the expression of self-renewal and invasion-related DEGs. RNA was prepared by Aurum™ total RNA Mini kit (Bio-Red, USA) following the manufacturer’s protocol. Then, the cDNA was synthesized using iscript™ cDNA synthesis kit (Bio-Red, USA). The qRT-PCR analysis was performed using Power SYBR® Green PCR Master Mix (Applied Biosystems, USA) on an ABI 7500 Fast Real-Time PCR System (Applied Biosystems, USA). Finally, the mRNA expression levels of target genes were quantified relative to GADPH and calculated using comparative cycle threshold (CT) (2^−ΔΔCT^) method. The sequences of PCR primers were listed in Table 1.

**Table 1:** The sequences of PCR primer

Genes	Sequences
GAPDH	F: TGCACCACCAACTGCTTAGC
	R: GGCATGGACTGTGGTCATGAG
EGR1	F: GTCCCCGCTGCAGATCTCT
	R: TCCAGCTTAGGGTAGTTGTCCAT
STAT3	F: ATGGCCCAATGGAATCAGC
	R: TTATTTCCAAACTGCATCAA
FGF2	F: TTCCTGCGCCTGATGTCC
	R: GGTTCAGTTTGGGTTGCTTGT
CA9	F: GCCTTTGAATGGGCGAGTG
	R: CCTTCTGTGCTGCCTTCTCATC
C-JUN	F: TCCCCCAGCTATCTATATGCAAT
	R: TCACAGCACATGCCACTTGA
HMOX1	F: GGCAGAGAATGCTGAGTTCA
	R: CCACATAGATGTGGTACAGG
CTGF	F: CTGCCTACCGACTGGAAG
	R: GAAGGTATTGTCATTGGTAACTC

### Western blot

Antibodies to ENO1 (Abcam, Cambridge, UK), p-4EBP1 (ABclonal, Boston, USA), p-S6K (ABclonal, Boston, USA), p-mTOR (CST, Danvers, MA, USA), mTOR (Proteintech, Chicago, USA), p-ACC (ABclonal, Boston, USA), p-AMPKα (ABclonal, Boston, USA), AMPKα (Proteintech, Chicago, USA), β-actin (CST, Danvers, MA, USA), HRP-conjugated goat anti-mouse IgG (Jackson, Pennsylvania, USA), and HRP-conjugated goat anti-rabbit IgG (Jackson, Pennsylvania, USA) were used in this study.

Whole-cell lysates were extracted from treated cells using RIPA lysis buffer. The proteins concentration was detected using Bicinchoninic acid (BCA) method according to the manufacturer’s protocol (Thermo Fisher Scientific, USA). Equal amounts of proteins (30 μg) from each sample were separated by SDS-PAGE and transferred onto polyvinylidene fluoride (PVDF) membrane (Millipore, USA), followed by blocking with 5% skim milk. Thereafter, PVDF membrane was probed with the indicated antibodies, followed by incubation with corresponding HRP-conjugated secondary antibodies using standard protocols. The bands were visualized by enhanced chemiluminescence kit (Millipore, USA) and were semi-quantified subsequent to normalization with the density of endogenous control using Bandscan software.

### Statistical analysis

All statistical analysis was performed with SPSS statistics version 17.0 (SPSS Inc., USA). Results were presented as mean ± standard error of the mean (SEM) of three separate experiments. Statistical significance between two groups was performed by Students’ *t* test. Differences were considered statistically significant when *p* < 0.05.

## Results

### ENO1 was identified as an antigen of mAb 12C7 in LCSCs

**Fig. 1:**
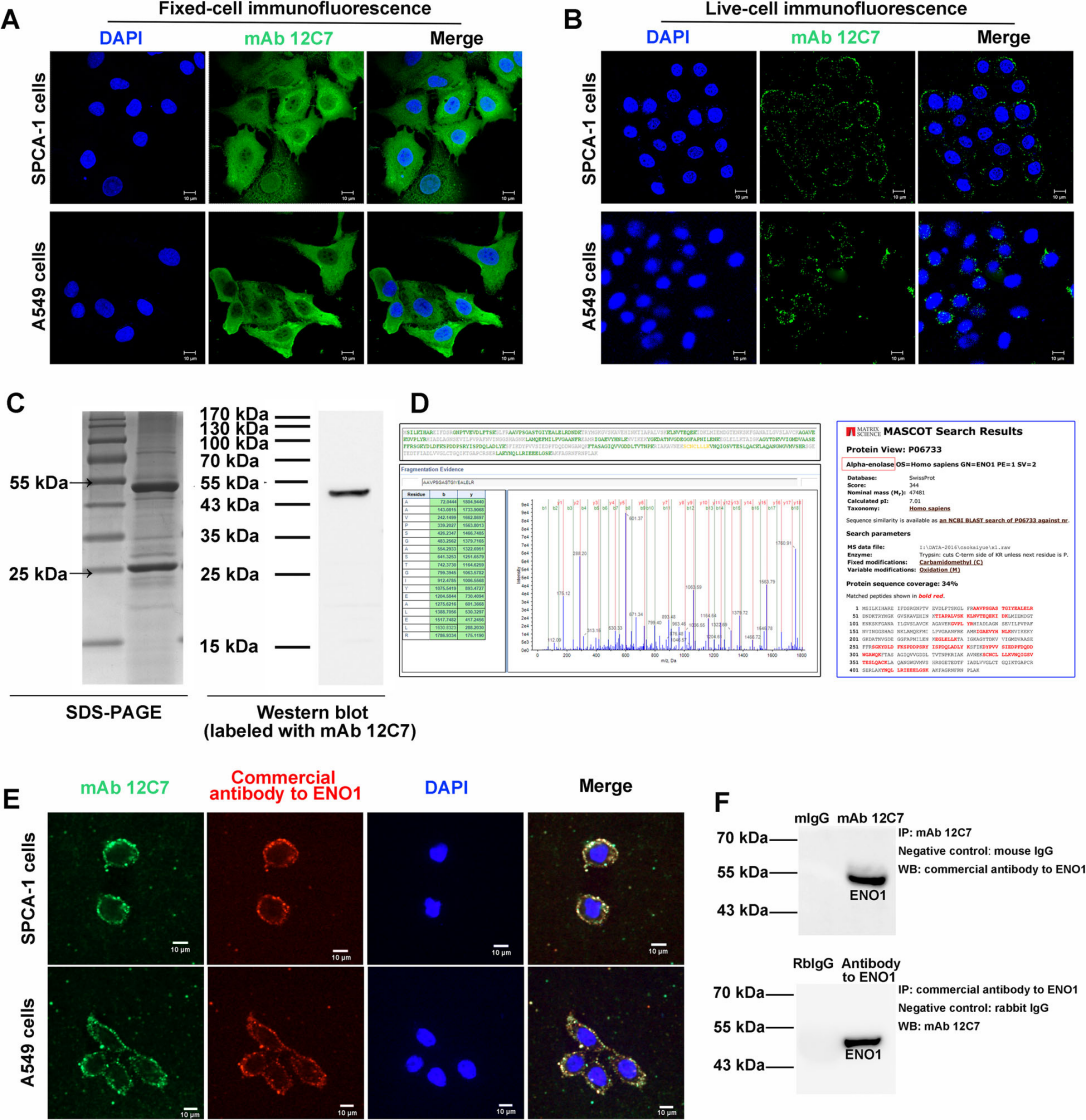
Localization and identification of targeted antigen recognized by mAb 12C7. **a** Fixed SPCA-1 and A549 sphere cells were labeled with mAb 12C7 (green) and counterstained with the nuclear stain DAPI (blue). Fluorescent microscope images (× 1000) showed that targeted antigen is abundantly localized in the membrane, cytoplasm and nucleus. Scale bar, 10 μm. **b** Live SPCA-1 and A549 sphere cells were labeled with mAb 12C7 (green), after fixing and washing, counterstained with the nuclear stain DAPI (blue). Images (× 1000) further verified the cell membrane immunoreactivity. Scale bar, 10 μm. **c** Purification of targeted antigen recognized by mAb 12C7. Purified antigen proteins recognized by mAb 12C7 from cell lysate of SPCA-1 sphere cells were electrophorized (right panel) and then immunoblotted with mAb 12C7 (left panel). Results presented a band of 43–55 kDa protein. **d** The 43–55 kDa protein band was excised from SDS-PAGE and identified as ENO1 by mass spectrometry (right panel) and analysis in Mascot database (left panel). **e** Immunofluorescence co-localization of targeted antigen in SPCA-1 and A549 cells. Cells were co-incubated with mAb 12C7 (green) and commercial antibody to ENO1 (red), and counterstained with the nuclear stain DAPI (blue). Fluorescent microscope images (× 1000) showed the high coincidence between antigens labeled with mAb 12C7 (green) and with commercial antibody to ENO1 (red). Scale bar, 10 μm. **f** Identification of mAb 12C7-binding antigen by immunoprecipitation-western blot. Immunoprecipitation was performed using purified mAb 12C7 or commercial antibody to ENO1, followed by western blotting against ENO1. Results confirmed that a 43–55-kDa protein band of ENO1 antigen interacting with mAb 12C7

In our previous study, we observed that mAb 12C7 targeted LCSCs and inhibited their biological properties [12]. Herein, the localization, purification, and identification of mAb 12C7 targeted antigen were performed in this study. Firstly, for targeted antigen localization, live-cell and fixed-cell immunofluorescence were respectively performed in A549 cells and SPCA-1 sphere cells using mAb 12C7. Immunofluorescence analysis showed that both in SPCA-1 and A549 sphere cells, the antigen recognized by mAb 12C7 were expressed on membrane, cytoplasm, and nucleus in fixed cell (Fig. 1a). Moreover, the live-cell immunofluorescence further verified the cell membrane immunoreactivity (Fig. 1b). Furthermore, in order to identify the specific antigen recognized by mAb 12C7, the cell lysate was purified by agarose G, and then electrophorized and immunoblotted with 12C7 antibodies in SPCA-1 sphere cells. As a result, a band of protein between 43 and 55 kDa was observed by Coomassie brilliant blue staining and confirmed by Western blotting (Fig. 1c). Then, the 43–55 kDa protein band was excised from SDS-PAGE for LC-MALDI-TOF/TOF mass spectrometry analysis. As data shown in Fig. 1d, the antigen recognized by mAb 12C7 was identified as human ENO1 by searching in Mascot database (Mascot score, 344; Estimated molecular weight/pI, 47,481/7.01; protein sequence coverage, 34%).

Subsequently, to further confirm ENO1 as the target antigen of mAb 12C7, the immunofluorescence co-localization and immunoprecipitation was performed using commercial antibody to ENO1 or the purified mAb 12C7. As shown in Fig. 1e, the target antigen labeled with mAb 12C7 (green fluorescence) was well-fitted to that labeled with commercial antibody to ENO1 (red fluorescence) and both co-located on the cell membrane. Moreover, immunoprecipitation also demonstrated that the 43–55 kDa protein band was either interacted with mAb 12C7 or commercial antibody to ENO1 (Fig. 1f). Taken together, these results indicated that ENO1 was the targeted antigen of mAb 12C7 in LCSCs.

### ENO1 promoted the self-renewal, growth, and invasion abilities of LCSCs

**Fig. 2:**
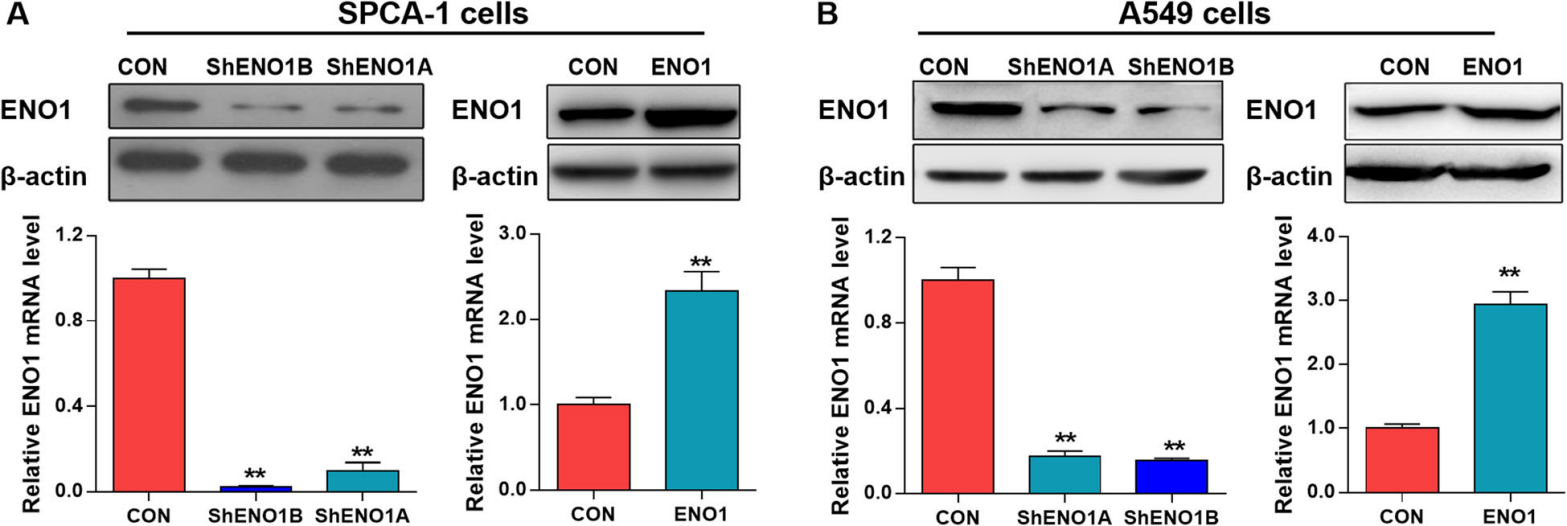
Abnormal expression of ENO1 was verified by Western blotting after transfection. **a** SPCA-1 and **b** A549 cells were respectively transfected with lentivirus silent vector (GV248-ShENO1B or GV248-ShENO1A) or overexpressed vector GV358-ENO1. Western blot analysis and qRT-PCR confirmed the downregulation or upregulation of ENO1 in two cells, respectively. Data are expressed as mean ± SEM of three independent experiments. **p < 0.01, compared to control (CON)

To investigate whether ENO1 participates in the biological properties of LCSCs, lentiviral transduction was performed to induce overexpression or silence of ENO1, respectively. Western blotting analysis and qRT-PCR confirmed the upregulation or downregulation of ENO1 in both SPCA-1 (Fig. 2a) and A549 cells (Fig. 2b) compared with the NC after lentivirus transfection. Notably, the downregulation of ENO1 by lentivirus vector GV248-ShENO1B was more significant than that by GV248-ShENO1A (Fig. 2a, b); thus, GV248-ShENO1B transfected cells were selected for the subsequent experiments.

**Fig. 3:**
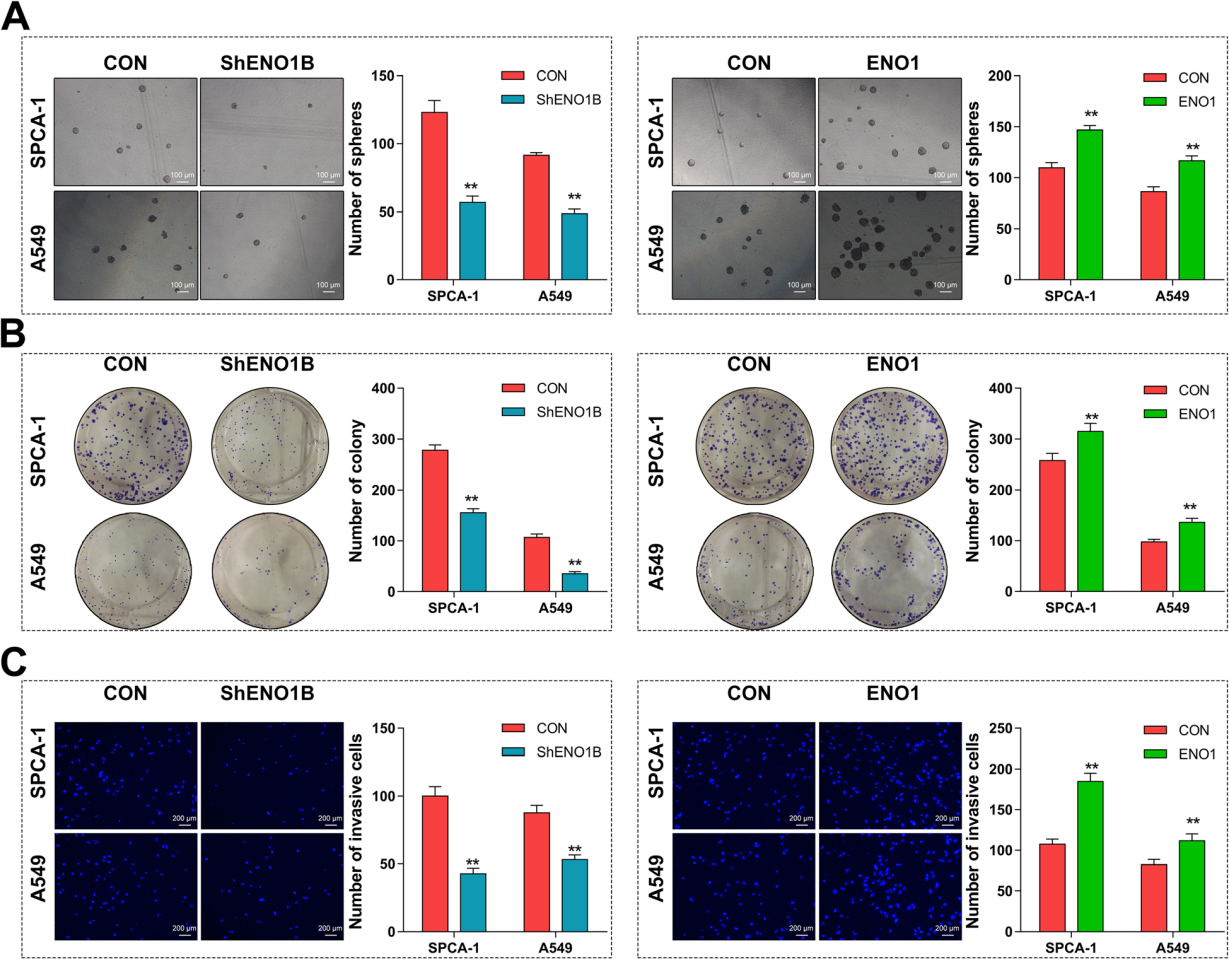
ENO1 promoted the self-renewal, growth, and invasion ability of LCSCs. Lung cancer stem cells (LCSCs) were enriched from GV248-ShENO1B or GV358-ENO1 transfected SPCA-1 and A549 cells. **a** Sphere formation assay demonstrated that the sphere formation abilities of two transfected LCSCs were both inhibited by ENO1 knockdown (right panel) but enhanced by ENO1 overexpression (left panel). Scale bar, 100 μm. **b** Colony formation assay demonstrated that the cell growth abilities of two transfected LCSCs were both inhibited by ENO1 knockdown (right panel) but enhanced by ENO1 overexpression (left panel). **c** Matrigel-coated Transwell assay demonstrated that the cell invasion ability of two transfected LCSCs were both inhibited by ENO1 knockdown (right panel) but enhanced by ENO1 overexpression (left panel). Scale bar, 100 μm. Data are expressed as mean ± SEM of three independent experiments. *p < 0.05, **p < 0.01, compared to control (CON)

Subsequently, LCSCs were enriched from transfected SPCA-1 and A549 cells for sphere formation, colony formation and invasion assay. As shown in Fig. 3a, comparing with the control, the sphere formation ability was remarkably inhibited by the downregulation of ENO1, while enhanced by the upregulation of ENO1 both in SPCA-1 and A549 sphere cells. Similarly, after lentivirus transfection, we observed that the growth and invasion ability of LCSCs was both significantly suppressed by ENO1 downregulation while promoted by ENO1 upregulation in two cells (Fig. 3b, c). Overall, these above results indicated that ENO1 promoted the self-renewal, growth, and invasion abilities of LCSCs.

### ENO1 was confirmed to regulate self-renewal and invasion-related gene in LCSCs by bioinformatics analysis

**Fig. 4:**
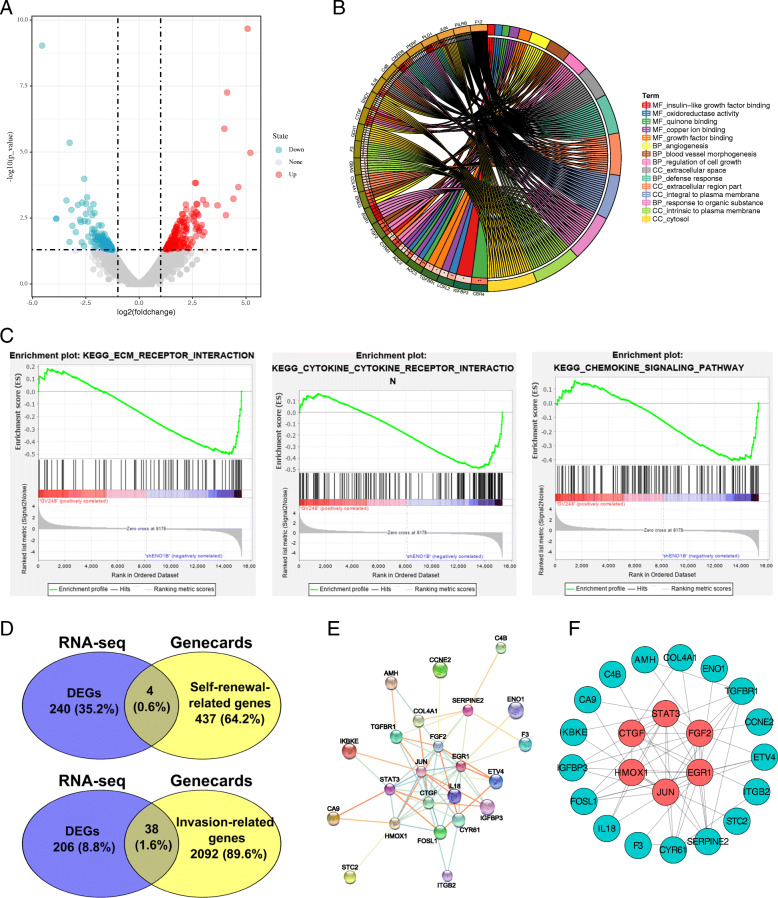
Bioinformatics analysis demonstrated that ENO1 regulates self-renewal and invasion-related gene in LCSCs. SPCA-1 sphere cells were transfected with GV248-CON and GV248-ShENO1B and collected for RNA-seq. Data were collected for bioinformatics analysis. **a** Volcano plot of the differentially expressed genes (DEGs) between the GV248-CON and GV248-ShENO1B transfected SPCA-1 sphere cells. Volcano plot showed the 244 DEGs, in which 92 were upregulated and 152 downregulated in ENO1-low expression cell compared to control. **b** GO analysis of DEGs showed the representative affected pathways. **c** GSEA plot showing ENO1 expression in association with extracellular matrix-receptor interaction, cytokine-cytokine receptor interaction, and chemokine signaling pathway. **d** Venny diagrams showed the screened DEGs, including 4 self-renewal-related DEGs and 38 invasion-related genes among 244 DEGs. **e** Network of invasion-related genes using STRING database. **f** Network of invasion-related genes using Cytoscape software. Top 6 nodes were highlighted in red

**Fig. 5:**
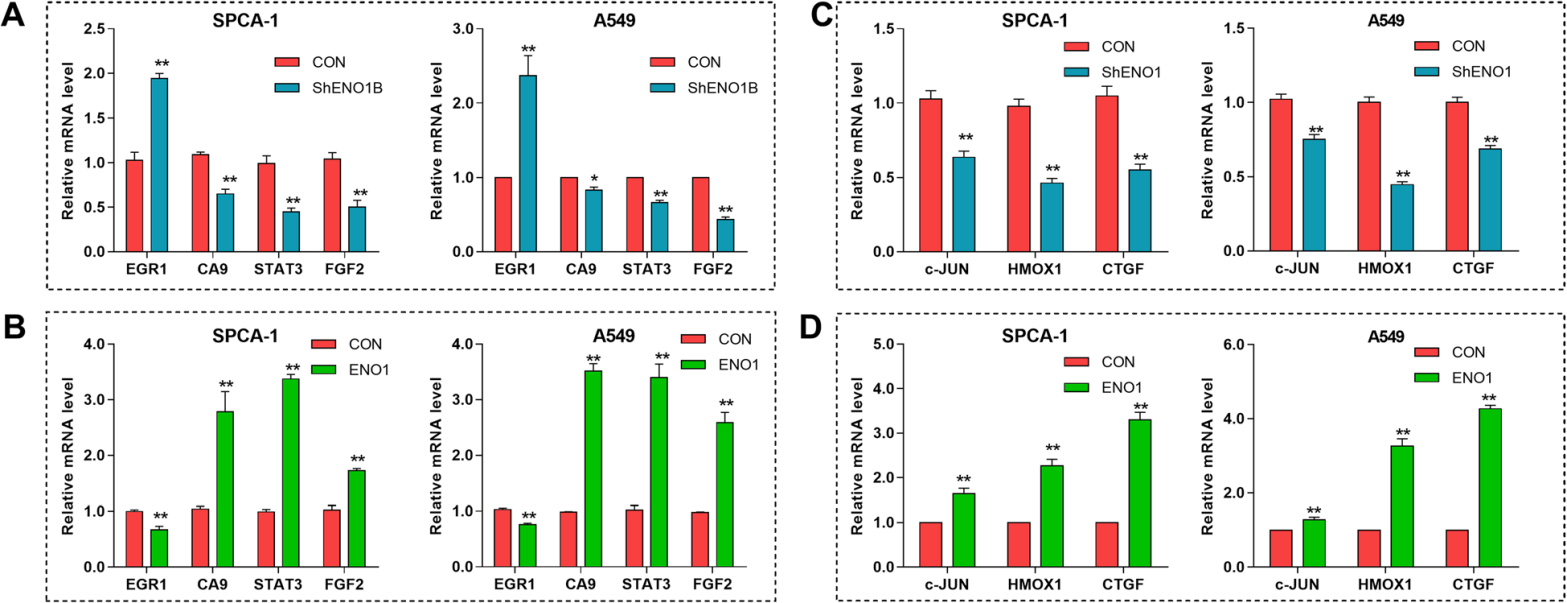
The qRT-PCR verification of screened DEGs. The expression of 4 self-renewal-related genes (EGR1, CA9, STAT3, FGF2) were verified by qRT-PCR in ENO1 knockdown (**a**) or overexpression (**b**), respectively. The expression of 3 invasion-related genes (c-JUN, HMOX1, CTGF) were verified by qRT-PCR in ENO1 knockdown (**c**) or overexpression (**d**), respectively. Results of qRT-PCR verification were consistent with bioinformatics analysis. Data are expressed as mean ± SEM of three independent experiments. *p < 0.05, **p < 0.01, compared to control (CON)

To examine the potential targets of ENO1 that could be involved in mediating the biological properties of LCSCs, SPCA-1 sphere cells were transfected with GV248-CON and GV248-ShENO1B and collected for RNA-seq to identify the DEGs. As a consequence, a total of 244 genes showed significantly differential expression. Among them, 92 genes were upregulated and 152 were downregulated in ENO1-low expression cell compared to control (Fig. 4a). GO analysis demonstrated that the DEGs showed significant enrichment of gene sets involved in the regulation of cell growth, angiogenesis, growth factor binding, apoptosis, and regulation of cell migration (Fig. 4b). Furthermore, GSEA plot showed that ENO1 expression was negatively correlated with the pathways related to cell self-renewal, migration, invasion, and metastasis-associated signaling, including extracellular matrix (ECM)-receptor interaction, cytokine-cytokine receptor interaction, and chemokine signaling pathway (Fig. 4c). Thus, to verify the role of ENO1 in self-renewal and invasion in lung cancer, we screened the self-renewal and invasion-related gene by using GeneCards database (http://www.genecards.org/) and Venny software. As shown in Fig. 4d, among 441 known self-renewal-related genes, a total of 4 self-renewal-related DEGs (*EGR1*, *CA9*, *STAT3*, *FGF2*) were found from these 244 DEGs. Thus, we confirmed their expression by qRT-PCR. As expected, we observed that ENO1 knockdown significantly upregulated *EGR1* and downregulated CA9, STAT3, and FGF2 (Fig. 5a), and completely opposite results induced by ENO1 overexpression both in SPCA-1 and A549 sphere cells (Fig. 5b).

Besides, by comparing with the known invasion-related genes via Venny software, we found 38 invasion-related genes among these 244 DEGs (Fig. 4d). Then, network analysis was performed for these invasion-related genes using STRING database and showed that a total of 23 genes had protein-protein interaction networks (Fig. 4e). Next, the networks were analyzed by Cytoscape software to obtain the connection values of each node. Eventually, the top 6 nodes (*EGR1*, *STAT3*, *FGF2*, *c-JUN*, *HMOX1*, *CTGF*) were determined in these 23 test networks (Fig. 4f). Here, due to the expression levels of *CA9*, *STAT3*, and *FGF2* have been detected, the remaining *JUN*, *HMOX1*, and *CTGF* were verified by qRT-PCR. The expression levels of c-JUN, HMOX1, and CTGF were decreased by ENO1 knockdown (Fig. 5c) and enhanced by ENO1 overexpression both in SPCA-1 and A549 sphere cells (Fig. 5d), which was consistent with the results of RNA-seq. Overall, we further confirmed the oncogenicity of ENO1 in LCSCs progression by bioinformatics analysis.

### Analysis of downstream pathway of ENO1 in LCSCs

**Fig. 6:**
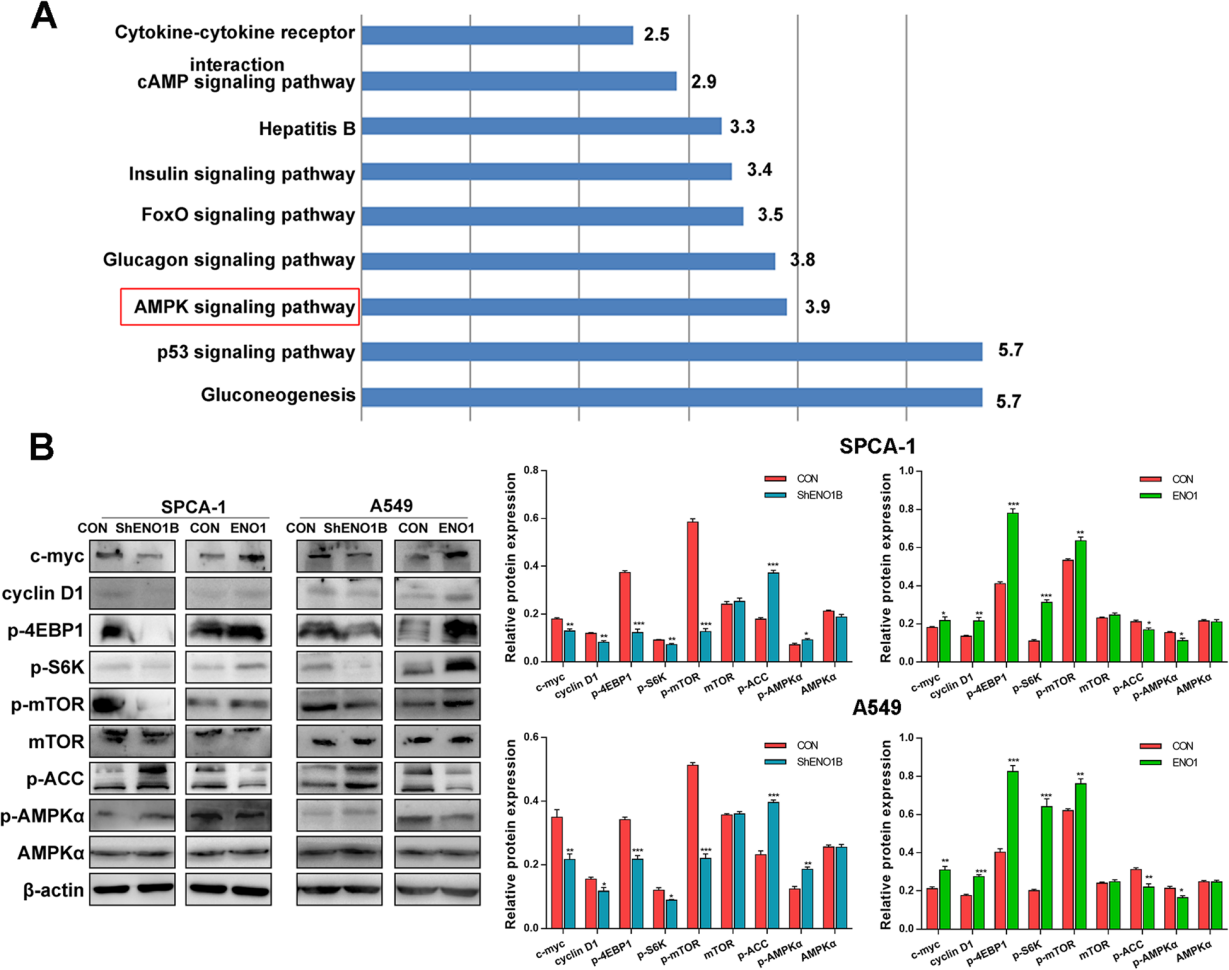
Analysis of downstream pathway of ENO1 in lung cancer. **a** KEGG pathway analysis of putative targets were shown. AMPK signaling pathway, as a central regulator of cellular and organismal metabolism, was selected for further confirmation. **b** Western blotting verified the expression of proteins-related to AMPK/mTOR pathway in ENO1 knockdown or overexpression sphere cells. Data are expressed as mean ± SEM of three independent experiments. *p < 0.05, **p < 0.01, compared to control (CON)

To further investigate the potential downstream pathways of ENO1 in LCSCs progression, KEGG pathway enrichment analysis of putative targets was performed. The results showed that the DEGs were enriched in multiple pathways as shown in Fig. 6a. Among them, AMPK signaling pathway, a master regulator of cellular metabolism, was verified in this study. Western blot assay revealed that the downregulation of ENO1 promoted the expression of p-AMPKα and p-ACC, but had no effect on the expression of total AMPKα, indicating the activation of AMPK signaling pathway (Fig. 6b). Meanwhile, the downregulation of ENO1 inhibited the phosphorylation of mTOR and its downstream transcription factors (S6K and 4EBP1), suggesting the inactivation of mTOR pathway (Fig. 6b). In addition, we found that the downregulation of ENO1 decreased the proto-oncogene c-myc and cyclin D1 expression (Fig. 6b). Conversely, the upregulation of ENO1 showed the completely opposite results, in which ENO1 inactivated AMPK pathway, activated mTOR pathway, and enhanced c-myc and cyclin D1 expression (Fig. 6c). Thus, we speculated that ENO1 may modulate the biological properties of LCSCs by AMPK/mTOR pathway to promote the progression of LCSCs.

## Discussion

Tumor-associated antigens (TAAs) are tumor-expressed antigenic proteins that are involved in cellular functions related to tumorigenesis and malignant transformation and thereby can be targeted in clinical cancer therapy [16]. Interestingly, wide-range evidences indicated that the autoantibodies can be used as probes to isolate, identify, and characterize potential TAAs [17]. In our previous, we successfully identified one mAb 12C7 and confirmed its anti-tumor activity [12]. Thus, based on our previous study, we here identified the putative targeted antigens using the purified mAb 12C7 and investigated the role of its targeted antigen in biological properties of LCSCs.

It is well-recognized that CSCs could express the TAAs, which can be recognized by autoantibodies [18, 19]. Identification and validation of TAAs in CSCs for autoantibodies is a crucial step in understanding their roles on a molecular basis in cancer immunology, as well as of interest for the advances in stem cells targeted therapies [20]. The 12C7, a novel mAb with anti-tumor activity in lung cancer, was developed by our laboratory [12]. However, we have not reported its anti-tumor mechanism and targeting in lung cancer. Herein, we firstly detected the localization of putative antigen recognized by mAb 12C7. As a consequence, the putative antigen was successfully recognized by 12C7 antibody and showed a surface antigen expression pattern, which was in accordance with the typical antigen expression pattern of CSCs [21]. Besides, ENO1 previously was reported to be expressed at the cell surface [22, 23], being consistent with our present observation that targeted antigen abundantly expressed at the cytomembrane of viable cell. Moreover, the abundant expression of ENO1 in membrane, cytoplasm, and nucleus in fixed cell implicated its great potential as a therapeutic target. In addition, the internalization antibody-antigen complexes would result in temporary or permanent disappearance of the antigens from the cell surface [24]. Here, the IF staining showed no obvious intracellular staining in live-cells, indicating that the internalization of 12C7 antibody-antigen complex is not significant. Thus, our finding further supported the hypothesis that ENO1 may be a potent therapeutic target. Subsequently, we found that the 43–55 kDa protein band purified from lung cancer sphere cells has a sequence coverage of 34% with human ENO1 and exists an interaction with 12C7 by immunoprecipitation, which was consistent with the result of commercial antibody to ENO1. Given these above evidence, the most striking finding of present study was that human ENO1 was identified as the targeted antigen of mAb 12C7 in LCSCs, providing a potential to improve the anti-tumor activity of 12C7.

ENO1 is a subtype of enolase, which is originally characterized as an enzyme involved in glycolytic metabolism [25]. Recently, ENO1 has been proved to perform pivotal role in the tumorigenesis of numerous cancers and acts as a good prognostic indicator to monitor the disease progression [26–29]. Because of this, ENO1 is emerging as a potential target in novel immunotherapies [22, 30]. CSCs hold stemness properties that sustain cancer progression, such as enhanced capacities for self-renewal cloning, growing, metastasizing, homing, and reproliferating [31]. Among them, the dysregulated self-renewal capacity is regarded as the defining feature of CSCs [32]. Targeting CSCs as a promising therapeutic strategy have aroused great interest in current researches [9]. Unfortunately, to our knowledge, there was no evidence of the function of ENO1 in LCSCs to date. Thereby, we innovatively studied the role of ENO1 in the biological properties, including self-renewal, growth, and invasion of LCSCs by lentivirus transduction. Sphere-forming assays have been widely used to evaluate self-renewal and differentiation at the single-cell level in vitro [33]. As expected, sphere-forming assays demonstrated that the self-renewal ability was remarkably inhibited by the downregulation of ENO1, while enhanced by the upregulation of ENO1 in LCSCs. Besides, the growth and invasion abilities of LCSCs were also enhanced by ENO1. Overall, ENO1 showed a powerful facilitation on in vitro self-renewal, growth, and invasion abilities of LCSCs. The self-renewal, growth, and invasion capacity of CSCs can drive malignant transformation [32], thus ENO1-mediated enhancement of these aggressive phenotypes of LCSCs would promote the lung cancer progression. Overall, this finding supported that ENO1 was an oncogene in lung cancer and emphasized the importance of ENO1 on stem cell maintenance, implicating a potential target for the stem cell therapy of lung cancer. Besides, our studies implicated that elaboration of the tumor-promoting action of ENO1 will lead to a better understanding of mAb 12C7-mediated anti-tumor mechanisms.

Although previous research revealed that ENO1 contributes to NSCLC progression by FAK/PI3K/AKT pathway [29], the specific mechanisms of ENO1 on regulating stem cell responses are still poorly understood. In this study, we explored its downstream targets/pathway using RNA-seq and bioinformatics analysis. Through bioinformatics analysis, we detected a total of 244 DEGs, which may be targeted by ENO1. Furthermore, the self-renewal-related genes (*EGR1*, *CA9*, *STAT3*, *FGF2*) and invasion-related genes (*EGR1*, *STAT3*, *FGF2*, *c-JUN*, *HMOX1*, *CTGF*) were found to be anomalously expressed in ENO1 knockdown cells by comparing the DEGs and Genecards database. Furthermore, these results were additionally confirmed by qRT-PCR analysis. Among them, EGR1, c-JUN, HMOX1, and CTGF were positively regulated by ENO1, while CA9, STAT3, and FGF2 were negatively regulated. Numerous studies have illuminated that the activation of CA9, STAT3, and FGF promotes the self-renewal capacity of CSCs [34–37]. Meanwhile, c-JUN, HMOX1, and CTGF play an oncogenic role in cancer [38–40]. Nevertheless, EGR1*,* a member of a zinc finger transcription factor family, has been described as tumor suppressor in cancer progression [41]. Additionally, GO analysis revealed that the ENO1-regulated DEGs participate in lots of key biological processes, such as cell growth and migration regulation, apoptosis, and angiogenesis. Meanwhile, GESA further confirmed that ENO1 negatively regulated the ECM-receptor interaction, cytokine-cytokine receptor interaction, and chemokine signaling pathway. These above pathways contribute to the cell invasion and cancer metastasis and provide signals that are essential for stem cell self-renewal [42–45]. Overall, the bioinformatics analysis and qRT-PCR validation both further supported the results of phenotypic study. Given these above evidence, we confirmed that ENO1 contributes to the self-renewal and invasion of LCSCs.

Additionally, we achieved a comprehensive understanding of the putative targets’ functions. KEGG analysis indicated that ENO1 might participate in several significant pathways to affect the stem cell maintenance, including gluconeogenesis, p53 signaling pathway, and AMPK signaling pathway. Among them, AMPK signaling pathway, as a central regulator of cellular and organismal metabolism, expressed ubiquitously in eukaryotic cells [46, 47]. AMPK affects many processes like gluconeogenesis, the phosphorylation of p53, and the activation of FoxO family transcription factors [48, 49]. Thereby, our present study mainly focused on the effects of ENO1 on AMPK signaling pathway. Western blot analysis demonstrated that ENO1 negatively regulated the phosphorylation of AMPKα and ACC, implicating that ENO1 inhibited the activation of AMPK signaling pathway. AMPK signaling pathway negatively regulates the mTOR pathway, promoting the cell growth [50]. Thus, we also measured the expression of proteins in mTOR pathway, its downstream transcription factor (4EBP1, S6K), and the growth related protein (cyclin D1 and c-myc). Not surprisingly, the phosphorylation of mTOR, 4EBP1, and S6K, and the expression levels of cyclin D1 and c-myc were remarkably enhanced by ENO1 overexpression. The mTOR stimulates the synthesis of some growth related proteins, such as cyclin D1 and c-myc, by phosphorylating 4EBP1and S6K [51]. Thus, the ENO1-mediated activation of mTOR pathway would contribute to the stem cell maintenance of lung cancer. In short, we speculate that ENO1, as an antigen of mAb 12C7, might promote the self-renewal, growth and invasion of LCSCs by AMPK/mTOR pathway. However, the proposed mechanism is only based on the changes in protein levels. Thus, the internal mechanism remains to be further researched adequately, for example the regulation of glycolysis. Given that the preliminary findings of glycolysis and ATP production regulated by ENO1, we would confirm whether ENO1 inactive the AMPK pathway by regulating the glycolysis levels, glucose uptake, lactic acid production, and the cellular ATP production. Moreover, the regulation of AMPK pathway by ENO1 would by validated using the inhibitor and activator of AMPK pathway, to support the findings from present study.

## Conclusion

The present study demonstrated that ENO1 is a targeted antigen of our novel developed mAb 12C7. More importantly, we revealed that ENO1 plays an oncogenic role in lung cancer by facilitating self-renewal, growth, and invasion of LCSCs. Besides, we preliminarily investigated the potential mechanism of ENO1-mediated tumor growth, that ENO1 might promote the biological properties of LCSCs by AMPK/mTOR pathway. These findings provide a potent therapeutic target for the stem cell therapy for lung cancer and have the potential to improve the anti-tumor activity of 12C7.

## Data Availability

The dataset supporting the conclusions of this article is included within the article.

## Abbreviations

TAAs: Tumor-associated antigens
mAb: Monoclonal antibody
LCSCs: Lung cancer stem cells
ENO1: α-Enolase
CSCs: Cancer stem cells
PBS: Phosphate buffer
BSA: Bovine serum albumin
LC-MALDI-TOF/TOF: Liquid chromatography-MALDI-tandem time-of-flight
DEGs: Differentially expressed genes
KEGG: Kyoto Encyclopedia of Genes and Genomes
SEM: Standard error of the mean
NSCLC: Non-small cell lung cancer

## References

[1] Torre LA, Siegel RL, Jemal A, Ahmad A, Gadgeel S (2016). Lung Cancer statistics. Lung cancer and personalized medicine: current knowledge and therapies.

[2] Siegel RL, Miller KD, Jemal A (2019). Cancer statistics, 2019. CA Cancer J Clin.

[3] Hirsch FR, Scagliotti GV, Mulshine JL, Kwon R, Curran WJ, Wu YL (2017). Lung cancer: current therapies and new targeted treatments. Lancet..

[4] Zappa C, Mousa SA (2016). Non-small cell lung cancer: current treatment and future advances. Transl Lung Cancer Res..

[5] Huang CY, Ju DT, Chang CF, Muralidhar Reddy P, Velmurugan BK (2017). A review on the effects of current chemotherapy drugs and natural agents in treating non-small cell lung cancer. BioMedicine..

[6] Janku F, Stewart DJ, Kurzrock R (2010). Targeted therapy in non-small-cell lung cancer--is it becoming a reality?. Nat Rev Clin Oncol.

[7] Lam S (2019). Lung cancer screening in never-smokers. J Thorac Oncol.

[8] Lundin A, Driscoll B (2013). Lung cancer stem cells: progress and prospects. Cancer Lett.

[9] Eramo A, Haas TL, De Maria R (2010). Lung cancer stem cells: tools and targets to fight lung cancer. Oncogene..

[10] Weiner GJ (2015). Building better monoclonal antibody-based therapeutics. Nat Rev Cancer.

[11] Ferris RL, Jaffee EM, Ferrone S (2010). Tumor antigen-targeted, monoclonal antibody-based immunotherapy: clinical response, cellular immunity, and immunoescape. J Clin Oncol.

[12] Cao K, Pan Y, Yu L, Shu X, Yang J, Sun L (2017). Monoclonal antibodies targeting non-small cell lung cancer stem-like cells by multipotent cancer stem cell monoclonal antibody library. Int J Oncol.

[13] Wu X, Chen H, Wang X (2012). Can lung cancer stem cells be targeted for therapies?. Cancer Treat Rev.

[14] Zhang X, Lou Y, Zheng X, Wang H, Sun J, Dong Q (2015). Wnt blockers inhibit the proliferation of lung cancer stem cells. Drug Des Devel Ther.

[15] Cho SB, Ahn KJ, Kim DH, Zheng Z, Cho S, Kang SW (2012). Identification of HnRNP-A2/B1 as a target antigen of anti-endothelial cell IgA antibody in Behcet's disease. J Invest Dermatol.

[16] Zhu Q, Liu M, Dai L, Ying X, Ye H, Zhou Y (2013). Using immunoproteomics to identify tumor-associated antigens (TAAs) as biomarkers in cancer immunodiagnosis. Autoimmun Rev.

[17] Liu W, Peng B, Lu Y, Xu W, Qian W, Zhang JY (2011). Autoantibodies to tumor-associated antigens as biomarkers in cancer immunodiagnosis. Autoimmun Rev.

[18] Codony-Servat J, Rosell R (2015). Cancer stem cells and immunoresistance: clinical implications and solutions. Transl Lung Cancer Res.

[19] Parmiani G, Maccalli C, Maio M (2015). Integrating immune checkpoint blockade with anti-neo/mutated antigens reactivity to increase the clinical outcome of immunotherapy. Vaccines (Basel).

[20] Imai K, Hirata S, Irie A, Senju S, Ikuta Y, Yokomine K (2011). Identification of HLA-A2-restricted CTL epitopes of a novel tumour-associated antigen, KIF20A, overexpressed in pancreatic cancer. Br J Cancer.

[21] Yi SY, Hao YB, Nan KJ, Fan TL (2013). Cancer stem cells niche: a target for novel cancer therapeutics. Cancer Treat Rev.

[22] Cappello P, Principe M, Bulfamante S, Novelli F (2017). Alpha-enolase (ENO1), a potential target in novel immunotherapies. Front Biosci (Landmark Ed).

[23] Capello M, Ferri-Borgogno S, Cappello P, Novelli F (2011). α-Enolase: a promising therapeutic and diagnostic tumor target. FEBS J.

[24] BANKA CL, CALARCO PG (2013). The immunological approach to the study of preimplantation mammalian. Manipulation Mammalian Dev.

[25] Zhou J, Zhang S, Chen Z, He Z, Xu Y, Li Z (2019). CircRNA-ENO1 promoted glycolysis and tumor progression in lung adenocarcinoma through upregulating its host gene ENO1. Cell Death Dis.

[26] Dai L, Qu Y, Li J, Wang X, Wang K, Wang P (2017). Serological proteome analysis approach-based identification of ENO1 as a tumor-associated antigen and its autoantibody could enhance the sensitivity of CEA and CYFRA 21-1 in the detection of non-small cell lung cancer. Oncotarget..

[27] Principe M, Borgoni S, Cascione M, Chattaragada MS, Ferri-Borgogno S, Capello M (2017). Alpha-enolase (ENO1) controls alpha v/beta 3 integrin expression and regulates pancreatic cancer adhesion, invasion, and metastasis. J Hematol Oncol.

[28] Cheng Z, Shao X, Xu M, Zhou C, Wang J (2019). ENO1 acts as a prognostic biomarker candidate and promotes tumor growth and migration ability through the regulation of Rab1A in colorectal Cancer. Cancer Manag Res.

[29] Fu Q-F, Liu Y, Fan Y, Hua S-N, Qu H-Y, Dong S-W (2015). Alpha-enolase promotes cell glycolysis, growth, migration, and invasion in non-small cell lung cancer through FAK-mediated PI3K/AKT pathway. J Hematol Oncol.

[30] Ray A, Song Y, Du T, Chauhan D, Anderson KC (2020). Preclinical validation of alpha-Enolase (ENO1) as a novel immunometabolic target in multiple myeloma. Oncogene..

[31] Aponte PM, Caicedo A. Stemness in Cancer: Stem Cells, Cancer Stem Cells, and Their Microenvironment. Stem Cells Int. 2017;2017:5619472.10.1155/2017/5619472. 10.1155/2017/5619472 PMC5394399 28473858

[32] O'Brien CA, Kreso A, Jamieson CHM (2010). Cancer stem cells and self-renewal. Clin Cancer Res.

[33] Pastrana E, Silva-Vargas V, Doetsch F (2011). Eyes wide open: a critical review of sphere-formation as an assay for stem cells. Cell Stem Cell.

[34] Coutu DL, Galipeau J (2011). Roles of FGF signaling in stem cell self-renewal, senescence and aging. Aging..

[35] Almiron Bonnin DA, Havrda MC, Lee MC, Liu H, Zhang Z, Nguyen LN (2018). Secretion-mediated STAT3 activation promotes self-renewal of glioma stem-like cells during hypoxia. Oncogene..

[36] Pastorek J, Pastorekova S (2015). Hypoxia-induced carbonic anhydrase IX as a target for cancer therapy: from biology to clinical use. Semin Cancer Biol.

[37] Ivanova L, Zandberga E, Silina K, Kalnina Z, Abols A, Endzelins E (2015). Prognostic relevance of carbonic anhydrase IX expression is distinct in various subtypes of breast cancer and its silencing suppresses self-renewal capacity of breast cancer cells. Cancer Chemother Pharmacol.

[38] Kappelmann-Fenzl M, Gebhard C, Matthies AO, Kuphal S, Rehli M, Bosserhoff AK (2019). C-Jun drives melanoma progression in PTEN wild type melanoma cells. Cell Death Dis.

[39] Capparelli C, Whitaker-Menezes D, Guido C, Balliet R, Pestell TG, Howell A (2012). CTGF drives autophagy, glycolysis and senescence in cancer-associated fibroblasts via HIF1 activation, metabolically promoting tumor growth. Cell Cycle.

[40] Yim M-S, Ha Y-S, Kim IY, Yun S-J, Choi YH, Kim W-J (2011). HMOX1 is an important prognostic indicator of nonmuscle invasive bladder cancer recurrence and progression. J Urol.

[41] Sakakini N, Turchi L, Bergon A, Holota H, Rekima S, Lopez F (2016). A positive feed-forward loop associating EGR1 and PDGFA promotes proliferation and self-renewal in glioblastoma stem cells. J Biol Chem.

[42] Xu L, Hou Y, Tu G, Chen Y, Du Y-E, Zhang H (2017). Nuclear Drosha enhances cell invasion via an EGFR-ERK1/2-MMP7 signaling pathway induced by dysregulated miRNA-622/197 and their targets LAMC2 and CD82 in gastric cancer. Cell Death Dis.

[43] Lukjanenko L, Jung MJ, Hegde N, Perruisseau-Carrier C, Migliavacca E, Rozo M (2016). Loss of fibronectin from the aged stem cell niche affects the regenerative capacity of skeletal muscle in mice. Nat Med.

[44] Yi L, Zhou X, Li T, Liu P, Hai L, Tong L (2019). Notch1 signaling pathway promotes invasion, self-renewal and growth of glioma initiating cells via modulating chemokine system CXCL12/CXCR4. J Exp Clin Cancer Res.

[45] Gilbert CA, Slingerland JM (2013). Cytokines, obesity, and cancer: new insights on mechanisms linking obesity to Cancer risk and progression. Annu Rev Med.

[46] Hardie DG, Schaffer BE, Brunet A (2016). AMPK: an energy-sensing pathway with multiple inputs and outputs. Trends Cell Biol.

[47] Mihaylova MM, Shaw RJ (2011). The AMPK signalling pathway coordinates cell growth, autophagy and metabolism. Nat Cell Biol.

[48] Carling D, Mayer FV, Sanders MJ, Gamblin SJ (2011). AMP-activated protein kinase: nature's energy sensor. Nat Chem Biol.

[49] Hardie DG, Ross FA, Hawley SA (2012). AMP-activated protein kinase: a target for drugs both ancient and modern. Chem Biol.

[50] Shaw RJ (2009). LKB1 and AMP-activated protein kinase control of mTOR signalling and growth. Acta Physiol.

[51] Inoki K, Corradetti MN, Guan K-L (2005). Dysregulation of the TSC-mTOR pathway in human disease. Nat Genet.

